# Quality of life instruments in atrial fibrillation: a systematic review of measurement properties

**DOI:** 10.1186/s12955-022-02057-y

**Published:** 2022-10-17

**Authors:** Alicia Sale, Jessica Yu

**Affiliations:** 1 Medtronic, Mounds View, MN USA; 2 Medtronic International Trading Sàrl, Tolochenaz, Switzerland

**Keywords:** Atrial fibrillation, Catheter ablation, Quality of life, Patient reported outcomes, Psychometrics

## Abstract

**Objectives:**

To identify the most frequently used atrial fibrillation-specific quality of life (QoL) instruments across atrial fibrillation (AF) ablation studies and to perform a systematic review of the most frequently used instrument’s measurement properties. This study uses quality of life instruments as an overarching term for any patient reported outcome measure that assesses a person’s health related well-being, functional status, and disease related symptoms.

**Methods:**

A literature mapping exercise was undergone to identify the most frequently used AF-specific QoL instruments across AF ablation studies published from 2016 to 2021. A systematic review of the most frequently used AF QoL instruments identified from the mapping exercise was performed using the COSMIN guidelines for systematic reviews of patient-reported outcome measurements. A systematic search was conducted in Ovid MEDLINE, Ovid Embase, Ovid PsycINFO, EBSCO CINAHL, and Cochrane CENTRAL. The search used variations of the keywords “atrial fibrillation”, “quality of life”, and “catheter ablation”.

**Results:**

Forty-five instruments were identified via the literature mapping exercise. After excluding non-patient reported outcome instruments, non-AF specific instruments, and instruments appearing only once, six instruments were identified: AF Effect on QualiTy-of-Life (AFEQT), AF Severity Scale, Minnesota Living with Heart Failure Instrument, AF Quality of Life Instrument, Arrhythmia Specific instrument in Tachycardia and Arrhythmia (ASTA), and SCL (Arrhythmia Symptom Checklist, Frequency and Severity). A systematic review of these six AF-specific health related QoL instruments was performed. We screened 3221 articles and 17 studies were eligible for inclusion. Using the COSMIN guidelines, ASTA and AFEQT had the best ratings across measurement properties with both instruments having good ratings for instrument development and internal consistency. However, none of the 17 included articles assessed measurement error and cross-cultural validity.

**Conclusions:**

AFEQT and ASTA had the strongest measurement properties but not all measurement properties were assessed. Considering the large number of indeterminate and insufficient ratings, future research should focus on cross-cultural validation, measurement error, responsiveness, and interpretability. This review summarizes the current evidence for AF QoL instruments across AF ablation studies and outlines areas for future research.

**Supplementary Information:**

The online version contains supplementary material available at 10.1186/s12955-022-02057-y.

## Introduction

Atrial fibrillation (AF) is the most common cardiac arrhythmia worldwide [1]. AF is a chronic and progressive disease caused by ineffective atrial contraction. Age is an important risk factor for AF and improvements in life expectancy has increased the AF incidence rate by 31% over the past 20 years [2]. Global AF prevalence will continue to increase if life expectancy continues to improve. As a result of its increasing prevalence, AF presents a significant economic burden to healthcare systems [3].

Patients with AF experience symptoms such as palpitations, chest tightness, fatigue, shortness of breath, and dizziness, all of which limit the ability to perform daily activities [1, 4]. Other AF related outcomes include stroke risk, heart failure, depression, and impaired quality of life [1]. QoL has been found to be lower in AF patients than in both healthy individuals and patients with other cardiovascular diseases [5, 6]. Current AF management, including rate and rhythm control, aims to improve QoL through symptom alleviation. Treatment options for rhythm control include cardioversion, anti-arrhythmic drugs (AADs), and catheter ablation [1]. AAD medical therapy is well established for AF patients however, catheter ablation has seen significant technological advancements and therefore represents a large proportion of the clinical studies [7].

Although the main objective of such therapies is QoL improvement, there is a lack of appreciation in how to measure patient QoL in clinical practice. In the context of AF, studies have found diagnostic ECG results to have a weak and inconsistent relationship with QoL impairment, suggesting that QoL outcomes in AF patients are not determined by clinical indicators alone [11]. QoL instruments can be generic or condition specific. Generic instruments are designed to be used across a wide range of conditions [8]. Condition-specific instruments are designed for a specific population, such as individuals with AF. Although not as generalizable as generic instruments, condition-specific instruments have greater sensitivity to detect small changes in QoL and may be less impacted by comorbidities unrelated to study interventions [9].

The primary objective of this study is to review the measurement properties of the most frequently used AF-specific health-related quality of life (QoL) instruments through a systematic review of the studies which validated and designed these instruments. This review used the COSMIN methodology guidelines for systematic reviews of patient-reported outcome measurements which includes rigorous assessment of validity, reliability, and instrument responsiveness.

## Methods

### Literature mapping exercise

Literature mapping exercises are used to characterize a large body of literature aiming to guide decisions about more focused analyses [10]. A literature mapping exercise was completed prior to our systematic review to identify the most common AF QoL instruments used across articles assessing catheter ablation for AF. This study uses quality of life instruments as an overarching term for any patient reported outcome measure (PROM) that assesses a person’s health related well-being, functional status, and disease related symptoms. Because previous reviews [11–13] have identified over 40 distinct AF QoL instruments, this exercise assisted with narrowing the scope for the systematic review.

A literature search was performed in Embase for articles published from 2016 to 2021, using the keywords “atrial fibrillation”, “quality of life”, and “catheter ablation”. The full search strategy can be found in Additional file 1: Appendix A. A five-year time frame was used to assess whether AF QoL instruments used across studies have changed since Kotecha et al. review [13] of AF QoL instruments was published in 2016 [13]. Catheter ablation was added as a keyword to restrict the type of intervention given the breadth of studies on ablation due to continual technological advancements and iterations and its primary objective to improve patients QoL.

Article titles and abstracts were screened by two reviewers. Studies including participants with heart failure (HF) or other arrhythmias were included if the population studied also had AF. HF was included because HF and AF coexist and share a bidirectional relationship [14]. AF occurs in more than half of individuals with HF and HF occurs in more than one third of individuals with AF [15]. Studies were included if they measured QoL outcomes and reported the AF QoL instruments used. Editorials, clinical guidelines, and abstract posters were excluded. AF QoL instruments cited more than once were included in the systematic review search strategy.

### Search strategy and selection criteria

To review the measurement properties of the AF-specific quality of life (QoL) instruments identified from the literature mapping exercise outlined above, a systematic review was undertaken to assess articles that validated or designed these instruments.

A systematic search was performed in Ovid MEDLINE, Ovid Embase, Ovid PsycINFO, EBSCO CINAHL, and Cochrane CENTRAL on July 30, 2021, for studies published in any language between inception to July 2021. The search strategy, which consisted of filters for AF, QoL instruments, and measurement properties, was derived from the COSMIN guidelines [16]. The COSMIN search strategies were translated for use in Ovid, EBSCO, and Cochrane interfaces, for use in this review. A copy of the full search strategy for MEDLINE can be found in Additional file 1: Appendix B.

We included all studies with a population of AF patients that appraised the measurement properties of one of the AF QoL instruments identified from the mapping exercise above. Only full text articles were included. Two reviewers independently screened titles and abstracts and conflicts were resolved through discussion between the two reviewers. The same process was repeated for full text eligibility screening.

### Data extraction

Data items included article title, author, year of publication, country, population characteristics, AF QoL instrument characteristics, measurement properties, and information on instrument interpretability and feasibility. Data extraction for included studies was completed by one reviewer. The second reviewer confirmed the entries by comparing the completed data extraction table with full text articles.

### Quality assessment and risk of bias

The COSMIN Risk of Bias checklist [17] was used to assess the methodological quality and risk of bias in each single study. The studies were rated as very good, adequate, doubtful, or inadequate quality according to the Risk of Bias checklist. Then, the results of each single study were rated according to the COSMIN criteria for good measurement properties, which are summarized in Table 1. The results were rated sufficient, insufficient, or indeterminate. Once each study was rated according to the COSMIN Risk of Bias checklist and the criteria of good measurement properties, the results from all included studies on an instrument were pooled together. The overall rating was then compared against the criteria of good measurement properties to arrive at a final rating of sufficient, insufficient, or indeterminate. Each criterion per measurement property was scored through a quality assessment to determine the quality score for each study. Quality assessment and risk of bias were completed independently by each reviewer with conflicts being resolved through discussion between the two reviewers.

**Table 1:** Definition and criteria of good measurement properties, as defined by the COSMIN guidelines [18]

Measurement property	Definition	Criteria (+ if)
*Validity*		
Structural validity	Degree to which PROM is an adequate reflection of the dimensionality of the construct to be measured	CFA: (CFI or TFLI or comparable measure > 0.95 OR RMSEA < 0.06 OR SRMR < 0.08
Hypotheses testing	Convergent or discriminant: degree to which expected similar domains between measurement tools are in fact similar or degree to which expected different domains between measurement tools are in fact different	≥ 75% in accordance with hypothesis
Cross‐cultural validity	Degree to which the performance of the items on a translated or culturally adapted PROM are an adequate reflection of the performance of the original PROM	No important differences found between group factors (such as age, gender, language) in multiple group factor analysis OR no important DIF for group factors (McFadden’s R^2^ < 0.02)
Criterion validity	The degree to which the scores of a PROM are an adequate reflection of a gold standard	Correlation with gold standard ≥ 0.70 OR area under curve (AUC) ≥ 0.70
*Reliability*		
Internal consistency	Degree of interrelatedness among the items in a PROM	Cronbach's alpha ≥ 0.70
Reliability	Proportion of the total variance in the measurements which is due to true different between patients (test–retest, inter–rater, or intra–rater)	Intraclass correlation coefficient (ICC) or weighted Kappa ≥ 0.70
Measurement error	The degree to which the scores of a PROM are attributed to true changes in the construct being measured	Smallest detectable change (SDC) or limits of agreement (LoA) < minimal important change (MIC)
*Responsiveness*		
Responsiveness	The ability of a PROM to detect change over time	The result is in accordance with the hypothesis OR area under curve (AUC) ≥ 0.70

*PROM* patient reported outcome measurement, *CFA* Confirmatory factor analysis, *CFI* comparative fit index, *TLI* Tucker-Lewis’s index, *RMSEA* root mean square error of approximation, *SRMR* standardized root mean squared residual

## Results

### Literature mapping exercise

**Fig. 1:**
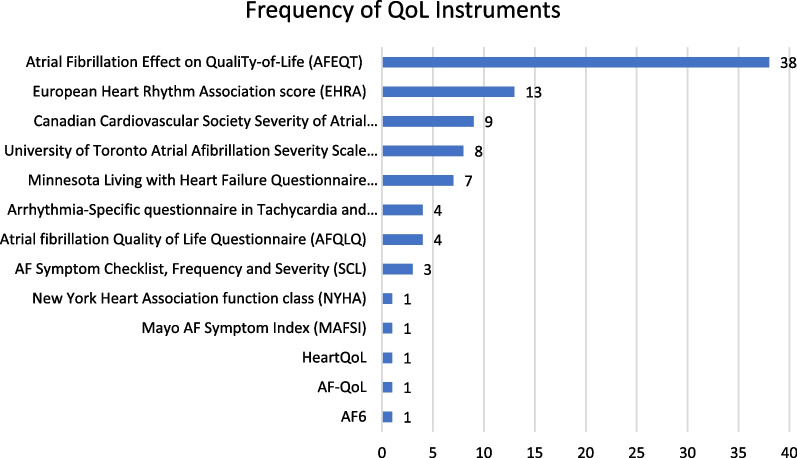
Frequency of use of AF QoL instruments across AF ablation studies

The literature mapping exercise identified 106 unique articles including 45 QoL instruments. 13 of these instruments were considered AF specific and are listed in Fig. 1. The EHRA and CCS-SAF scales were excluded from the systematic review because they are caregiver evaluated scores rather than patient reported. After this exclusion and the exclusion of instruments that only appeared in a single study, six instruments were included in the systematic review: AFEQT, AFSS, MLHF-Q, ASTA, AFQLQ, and SCL. A list of all 45 instruments identified from the mapping exercise can be found in Additional file 1: Appendix C.

### Systematic review: study selection

**Fig. 2:**
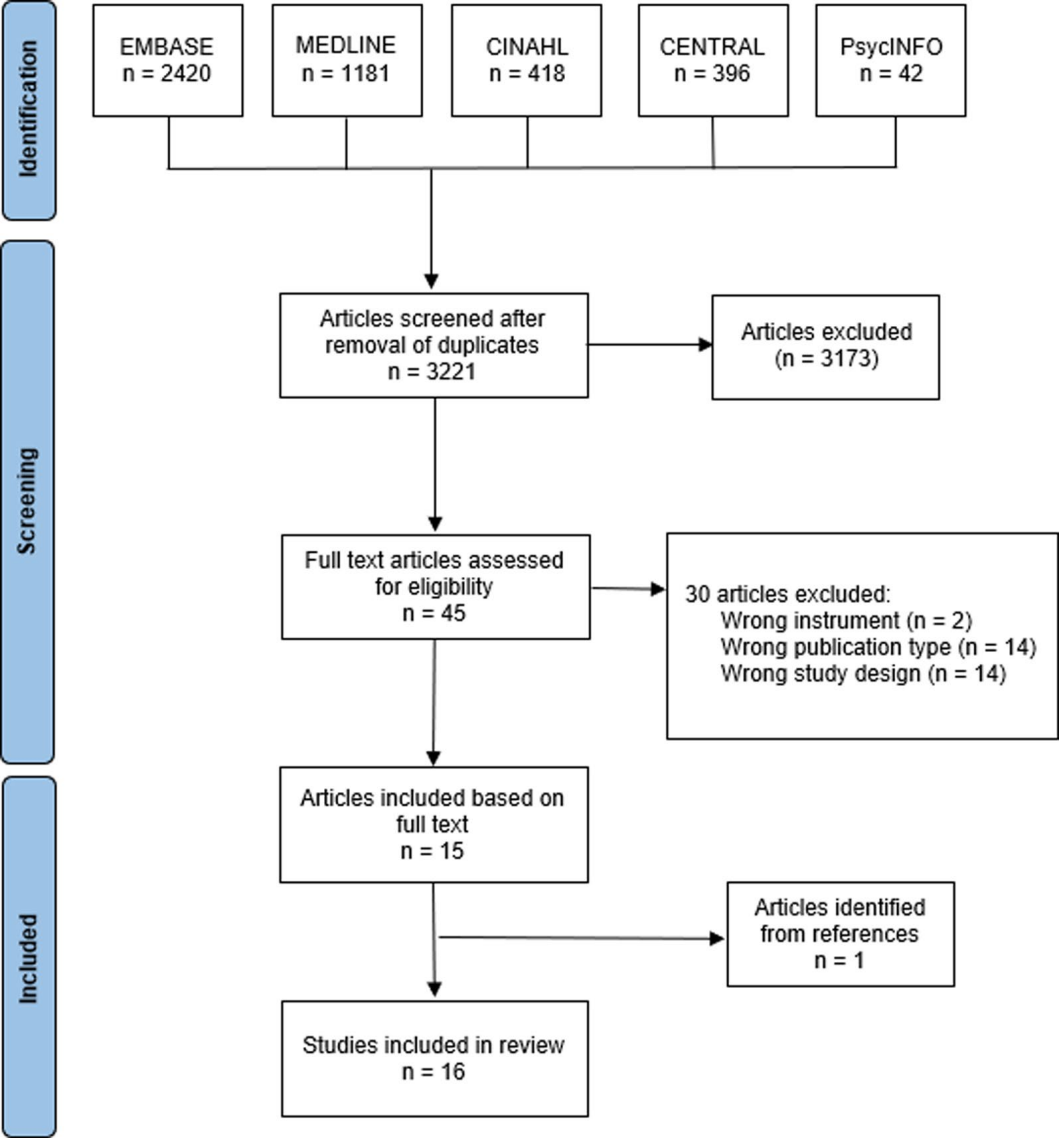
PRISMA flow diagram

The six AF QoL instruments identified from the literature mapping exercise were included and evaluated in our systematic review (AFEQT, AFSS, MLHF-Q, ASTA, AFQLQ, and SCL). The search results are outlined in the PRISMA flowchart in Fig. 2. After the removal of duplicates and the screening of full text articles, 16 studies were included in the review. One study was found from the reference list of an included article. All other articles were identified in the initial literature search.

### Characteristics of included studies

Table 2 provides a summary of the study characteristics of the included studies [19–35]. Table 3 summarizes the instrument characteristics for the six included QoL instruments.

**Table 2:** Study characteristics of the included studies

Instrument	Authors	Years	Country	Language	N	Mean age	Gender (% female)	Type of AF (%)
AFEQT [39]	Spertus et al.	2011	USA, Canada	English	214	62 ± 11.9	42.50%	66% paroxysmal, 24% persistent, 5% longstanding persistent, 5% permanent
AFEQT [40]	Dorian et al.	2013	USA, Canada	English	214	62 ± 11.9	42.50%	66% paroxysmal, 24% persistent, 5% longstanding persistent, 5% permanent
AFEQT [41]	Tailachidis et al.	2016	Greece	Greek	102	70 ± 9.2	46.10°%	59.8% permanent; 31.4% persistent; 8.8% paroxysmal
AFEQT [42]	Holmes et al.	2019	USA	English	1347	74 ± 9.8	43°%	8.2% first detected; 48.8% paroxysmal; 13.4% persistent; 29.6% permanent
AFEQT [43]	Gune§ et al.	2021	Turkey	Turkish	204	71.33 ± 10.34	65.2	88.2% permanent
AFEQT [44]	Li et al.	2021	Hong Kong	Chinese	200	69.8 ± 5.2	48.50%	44.5% paroxysmal; 52.5% undifferentiated; 1% persistent; 1% permanent
AFSS[45]	Dorian et al.	2002	Canada	English	161	58 ± 12	31°%	54% paroxysmal; 35% persistent; 11% permanent
AFSS[46]	Kahya Eren et al.	2014	Turkey	Turkish	130	63.1 ± 10.9	41.50%	30% paroxysmal; 6.2% persistent; 63.8% permanent
SCL [38]	Berkowitsch et al.	2003	Germany	N/A	60	58 ± 11	35%	100% paroxysmal refractory to > 3 AADs
SCL[47]	Bubien et al.	1996	USA	English	159	49 ± 15.8	56%	13.8% atrial fibrillation; 86.2% other arrhythmias
SCL [48]	Carnlof et al.	2020	Sweden	Swedish	646	61.4 ± 1.5	50%	52.3% atrial fibrillation; 47.7% other arrhythmias
AFQLQ [49]	Moreira et al.	2016	Brazil	English	40	61.2 ± 9.6	35%	47.5% persistent; 52.5% permanent
ASTA [50]	Walfridsson et al.	2015	Sweden	Swedish	270	59.3 ± 13	34%	67% AF; 33% other arrhythmias
ASTA [51]	Lomper et al.	2019	Poland	Polish	244	70.7 ± 10.7	56.60%	47.5% paroxysmal; 52.5% permanent/persistent
ASTA [52]	Cannavan et al.	2020	Brazil	Portugese	140	57.2 ± 13.1	55%	50.71% AF; 49.29% other arrhythmias
MLHF-Q [53]	Middel et al.	2001	Netherlands	Dutch	60	61.5 ± 12.7	35%	Type of AF not specified

**Table 3:** Characteristics of included QoL instruments

Instrument	Years	Therapeutic area	Constructs	Subscales/number of items	Response options	Original language	Available translations
*Specific*							
MLFH-Q [56]	1984	Heart failure	HRQoL	21 items; 3 domains: physical, emotional, overall health	Likert scale	English	50 + translations; Dutch translation included in review
SCL [47]	1996	Arrhythmia	Symptom severity, frequency	16 items; 2 domains	Likert scale	English	Swedish translation included in review
AFSS [45]	1998	AF	AF burden, health care utilization, symptoms	19 items; 3 parts: AF burden, health care utilization, symptom severity	Likert scale	English	Turkish translation included in review
AFQLQ [49]	2010	AF	Symptoms, daily activities, mental anxiety	30 items; 7 domains: palpitation, dyspnea, chest pain, dizziness, fatigue, well-being, illness perception	Likert scale	Portuguese	N/A
AFEQT [57]	2011	AF	HRQoL	20 items; 3 domains: daily activities, treatment concerns, symptoms, treatment satisfaction (not included in total score)	Likert scale	English	24 translations; Chinese, Turkish, Greek included in review
ASTA [50]	2015	Arrhythmia	HRQoL	13 items; 2 domains: physical and mental	Likert scale	Swedish	English, Polish, Portuguese

### Overview of QoL instruments

Outlined below is a concise overview of the results from our systematic review for each AF QoL instrument. The COSMIN risk of bias checklist and COSMIN criteria for good measurement properties were used to assess the study methodology and measurement property quality [17, 18]. The risk of bias and measurement property ratings can be found in Additional file 1: Appendix D. The data extraction table of measurement properties can be found in Additional file 1: Appendix E.

#### AFEQT

With six studies, AFEQT was the most validated QoL instrument in this systematic review. Three studies validated the translation of AFEQT into another language [23, 25, 26]. AFEQT also had the most comprehensive evaluation of interpretability [22, 24]. Dorian et al. [22] found a 19 point change in AFEQT score to represent a moderate improvement in QoL whereas Holmes et al. [24] found a 5 point change in AFEQT score to represent a clinically important difference. Spertus et al. [21] found that the question about sexual relationships had a disproportionately high missing response rate (15%). In comparison to generic instruments (EQ-5D and SF-36), AFEQT was found to show greater effect sizes for responsiveness, comparable to other condition-specific instruments like SCL and AFSS [21]. Many of the AFEQT measurement properties were indeterminate, either due to correlation coefficients not meeting the COSMIN criteria or missing statistical methods. The methodological quality of its content validity is unknown because it is unclear whether professionals were asked about AFEQT’s relevance or comprehensiveness during the development process.

#### AFSS

AFSS was evaluated in two studies [27, 28]. AFSS is considered a symptom scale rather than an overall QoL instrument because it only includes AF burden, symptoms, and healthcare utilization domains. The average completion time was less than 5 min [28]. AFSS is the only instrument to have insufficient internal consistency. Cronbach’s alpha for the healthcare utilization domain was less than 0.70, indicating that it is not closely related to the symptom and burden domains. Furthermore, hypothesis testing was also insufficient because it did not meet the correlation coefficient criteria. Test–retest reliability was found to be superior in AFSS, compared to all other QoL instruments [27]. Missing response rates varied from 0 to 7% across all items [28].

#### SCL

The SCL was evaluated in three studies [20, 29, 30]. Developed in 1996, it is the oldest instrument included in this study. Like the AFSS, the SCL is a symptom scale and it is used across all arrhythmias, not just AF. Although the SCL has been used for over two decades, the original English version has not been extensively validated. There are no published studies on the PROM development process or content validity. Structural validity was rated indeterminate because the required statistical measures were not reported. In terms of interpretability, response rates ranged from 94 to 84% [30].

#### AFQLQ

AFQLQ was only evaluated in one study [31] and the origins of it are unclear. It is likely to have originated in Brazil with translations mostly being done in languages other than English. The methodological quality used to calculate reliability is unclear as statistical methods were not reported. AFQLQ is the longest condition-specific instrument in this review with 30 questions, raising concerns about feasibility and completion time. Interpretability and feasibility characteristics were not reported.

#### ASTA

ASTA was evaluated in three studies [32–34], two of which were validating Polish and Portuguese translations. ASTA is the newest instrument included in this study and can be used in all types of arrhythmias. The content validity is unclear because it is reported whether patients were asked about the relevance of each individual item in the instrument. There was also uncertainty in hypothesis testing because correlation coefficients did not meet the COSMIN criteria. Otherwise, ASTA has relatively good measurement properties. The completion time was described as “a few minutes” [32]. Overall, 46% participants responded with “I don’t know” to at least one item, which increased the number of missing responses [32].

#### MLHF-Q

MLHF-Q was only evaluated in one study [35]; however, it has been validated in other populations outside of AF [36–38]. The justification for including MLHF-Q despite it being developed for HF was provided in the “Methods” section. Test–retest reliability was rated doubtful because the time interval between the two tests was 3 months, which is extensive for a reliability measurement. Structural validity was inadequate because the factor analysis sample size was too small. Hypothesis testing was rated insufficient because the correlation coefficients did not meet the COSMIN criteria. Interpretability was assessed through floor and ceiling effects, which found a moderate floor effect in 11.4–13.6% of scores.

### Synthesis of results

**Fig. 3:**
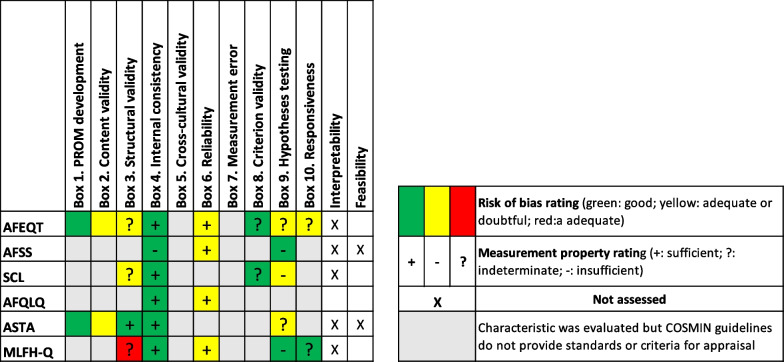
Synthesis of results

Results from the risk of bias and measurement property appraisals are qualitatively summarized in Fig. 3. Though not considered to be measurement properties, columns for interpretability and feasibility were added because they are important characteristics of QoL instruments [18]. Evidence was synthesized using the COSMIN guidelines. This final rating represents the psychometric quality of each instrument, taking into consideration the quality of measurement properties, study methodology, and risk of bias.

Our systematic review illustrated that none of the 16 studies evaluated the measurement properties of cross-cultural validity or measurement error. While multiple studies validated the translation of AF-related QoL instruments [25, 26, 28, 30, 33, 34], they did not complete cross-cultural validity tests as recommended by the COSMIN guidelines, which requires the inclusion of two subgroups for analysis [18]. Internal consistency was the only measurement property evaluated across all studies and was attributed the highest quality results (Fig. 2). PROM development and content validity were only assessed by the articles that originally developed the AF-related QoL instrument [21, 32]. Overall, ASTA and AFEQT were the two strongest performing AF-related QoL instruments in terms of measurement properties and study methodology, with sufficient ratings in both instrument development and internal consistency.

## Discussion

This comprehensive systematic review identified 6 AF-related QoL instruments and evaluated their measurement properties using the COSMIN guidelines. This highlights that, within the field of AF ablation clinical research, QoL instruments are constantly evolving, and new instruments are still being developed. The results of the literature mapping exercise are aligned with a previous study examining AF QoL instrument frequency [39]. Moreover, even though Coyne et al. [39] study was published in 2005, SF-36 was also the most common QoL instrument, followed by the SCL, NYHA score, and MLHF-Q appeared in both their study and this study’s mapping exercise [40].

Although this review identified different QoL instruments, the findings of this review are consistent with previous reviews [11–13]. Measurement error, cross-cultural validation, and responsiveness studies are still the most deficient areas of research and AFEQT is still the strongest rated AF QoL instruments. Moreover, even though an increasing number of psychometric studies on AF QoL instruments have been published in recent years, none of the available instruments have been fully validated across all measurement properties.

Responsiveness represents the ability of a QoL instrument to detect changes over time. A more responsive instrument will be able to detect smaller changes pre- and post-intervention. Interpretability is the degree to which one can assign meaning to a QoL instrument score or a change in scores. Measures like clinically important difference (CID) or minimally important change (MIC) are important for interpreting whether a change in score has a meaningful or significant impact on a patient’s QoL.

Interpretability is rarely measured for QoL instruments and even when measured, studies often produce conflicting results. In this systematic review, two studies assessed the interpretability of AFEQT. Dorian et al. [22] found a 19-point change in AFEQT score to represent a moderate improvement in QoL whereas Holmes et al. [24] found a 5-point change in AFEQT score to represent a CID. The large difference in scores could be attributed to the study authors using different anchors. For example, Dorian et al. used patient and physician assessments of QoL change and Holmes et al. used the EHRA score. Additionally, in a study calculating MIC using five different statistical methods, the five methods produced five different MIC values [44]. Our review outlines that further research is required in this area of psychometrics to standardize the statistical methods used to assess interpretability.

Hypothesis testing refers to the degree to which expected similar instruments are in fact similar or the degree to which expected dissimilar instruments are indeed dissimilar. Hypothesis testing is typically completed by calculating the correlation of scores from two presumably similar or dissimilar instruments. The hypothesis testing rating was determined by comparing the study results to the authors’ pre-determined hypothesis. Figure 2 illustrates that, nearly every AF QoL instrument in this review was rated poorly for hypothesis testing because the correlation coefficients were not significant enough to meet COSMIN criteria. This is anticipated considering that most hypothesis tests were completed with a condition-specific instrument and a generic instrument (e.g. correlation between AFSS and SF-36 scores) [41]. Since generic instruments are less sensitive to AF-related QoL compared to AF-specific instruments, weak correlations are to be expected [9].

The study that provided the best hypothesis testing result was Cannavan et al. [34]. Cannavan et al. [34] differed from the other studies because correlations were calculated for ASTA and AFQLQ scores, two AF/arrhythmia specific instruments, which yielded very strong correlations. Furthermore, Cannavan et al. [42] used Portuguese versions of ASTA and AFQLQ, with Portuguese being the original language of the AFQLQ. Results from Cannavan et al. [34] pose the question of whether there should be a universal AF-specific QoL instrument with translations or if each country or geographic region should develop their own instrument that is best suited for the setting. This could not be answered in this review because none of the included studies evaluated cross-cultural validity. In addition to responsiveness and interpretability, cross-cultural validation is another area that requires future research.

The findings of this review should be interpreted with consideration due to some limitations. Firstly, only condition specific AF-related QoL instruments were reviewed and generic QoL were out of scope [43, 41]. This is a limitation as it does not include all possible QoL instruments that could be used in an AF patient population. There are also some limitations to the use of the COSMIN guidelines. The COSMIN guidelines provide very high standards for what constitutes a good measurement property and good study methodology. To have good ratings across all measurement properties, many psychometric studies must be performed and published, which may not be feasible for every existing instrument. This suggests that there may be very well-developed instruments that exist but have yet to be psychometrically validated. While the COSMIN criteria for good measurement properties may be difficult to fulfil, it provides a benchmark for the development of new or updated instruments.

With the growing prevalence of AF QoL assessment is crucial. As outlined above this study identified four additional AF QoL instruments expanding on Kotecha 2016 review [13]. In addition, over 34 different QoL instruments have been leveraged across published studies (10). This emphasizes the lack of consensus on the most appropriate AF QoL instrument to use with patients. Our systematic review using COSMIN methodology suggests more robust validation is required. It should be noted that measurement properties are only one determinant of applicability of AF QoL instruments. Ease of use, administration, time taken, recall period and patient satisfactoriness are also important variables.

## Conclusion

This review identified six most frequently used AF- specific QoL instruments across AF ablation studies. Using the systematic COSMIN methodology, we undertook a review of these six AF- QoL instruments measurement properties through evaluating studies which validated and designed these instruments. We identified ASTA and AFEQT as the best validated instruments. However, further research is needed in areas of cross-cultural validation, measurement error, responsiveness, and interpretability across all six instruments.

## Supplementary Information

**Additional file1**. **Appendix A**: Embase search strategy for literature mapping exercise. **Appendix B**: Ovid MEDLINE search strategy for systematic review. **Appendix C**: Full results from literature mapping exercise. **Appendix D**: Risk of bias and measurement property appraisal results. **Appendix E**: Data extraction table of measurement properties.

## Data Availability

Data available on request.

## Abbreviations

AF: Atrial fibrillation
AFEQT: Atrial fibrillation effect on QualiTy-of-Life
AFQLQ: Atrial fibrillation quality of life questionnaire
AFSS: Atrial fibrillation severity scale
ASTA: Arrhythmia specific questionnaire in tachycardia and arrhythmia
CCS-SAF: Canadian Cardiovascular Society Severity of Atrial Fibrillation
CID: Clinically important difference
COSMIN: Consensus-based standards for the selection of health measurement instruments
EHRA: European Heart Rhythm Association
ESC: European Society of Cardiology
HF: Heart failure
HRQoL: Health-related quality of life
HTA: Health technology assessment
MIC: Minimally important change
MLHF-Q: Minnesota living with heart failure questionnaire
NYHA: New York Heart Association
PRO: Patient-reported outcome
PROM: Patient-reported outcome measure
QALY: Quality adjusted life year
QoL: Quality of life
SCL: Arrhythmia symptom checklist, frequency and severity
SDM: Shared decision making
SF-36: Medical Outcomes Study Short Form Health Survey
