# Supplementary material for: Quality of life instruments in atrial fibrillation: a systematic review of measurement properties
Source: Health Qual Life Outcomes. 2022 Oct 17;20:143. doi: 10.1186/s12955-022-02057-y (PMC9575307; doi:10.1186/s12955-022-02057-y)
Supplement: Supplementary file 1 — Additional file1. Appendix A: Embase search strategy for literature mapping exercise. Appendix B: Ovid MEDLINE search strategy for systematic review. Appendix C: Full results from literature mapping exercise. Appendix D: Risk of bias and measurement property appraisal results. Appendix E: Data extraction table of measurement properties. [file 12955_2022_2057_MOESM1_ESM.docx]

**APPENDIX A: Embase search strategy for literature mapping exercise**

Embase <2016 to 2021 July 16>

1 atrial fibrillation/

2 atrial fibrillat*.tw.

3 atrium fibrillat*.tw.

4 auricular fibrillat*.tw.

5 1 or 2 or 3 or 4

6 quality of life/

7 (hql or hqol or QoL).mp.

8 health-related quality of life.mp.

9 qol.mp.

10 quality of life.mp.

11 6 or 7 or 8 or 9 or 10

12 catheter ablation/

13 cardi* ablat*.tw.

14 (catheter adj6 ablat*).tw.

15 (cardi* adj6 ablat*).tw.

16 12 or 13 or 14 or 15

17 5 and 11 and 16

18 exp animal/ or exp animal experiment/ or nonhuman/

19 exp human/ or exp human experiment/

20 18 not 19

21 17 not 20

22 limit 21 to (english language and yr="2016 -Current")

23 editorial/

24 22 not 23

**APPENDIX B: Ovid MEDLINE search strategy for systematic review**

Ovid MEDLINE(R) ALL <1946 to July 29, 2021>

1 (HR-PRO or HRPRO or HRQL or QoL or QL or QoL).tw. or quality of life.mp. or (health index* or health indices or health profile*).tw. or health status.mp. or ((patient or self or child or parent or carer or proxy) adj (appraisal* or appraised or report or reported or reporting or rated or rating* or based or assessed or assessment*)).tw. or ((disability or function or functional or functions or subjective or utility or utilities or wellbeing or well being) adj2 (index or indices or instrument or instruments or measure or measures or instrument* or profile or profiles or scale or scales or score or scores or status or survey or surveys)).tw. 825853

2 instrumentation/ or methods/ or Outcome Assessment, Health Care/ or outcome assessment.tw. or outcome measure*.tw. or Health Status Indicators/ or Validation Studies.pt. or Comparative Study.pt. or psychometrics/ or psychometr*.tw. or clinimetr*.tw. or clinometr*.tw. or observer variation/ or observer variation.tw. or reproducibility of results/ or reproducib*.tw. or discriminant analysis/ or reliab*.tw. or unreliab*.tw. or valid*.tw. or coefficient of variation.tw. or coefficient.tw. or homogeneity.tw. or homogeneous.tw. or internal consistency.tw. or (cronbach* adj3 (alpha or alphas)).tw. or (item adj3 (correlation* or selection* or reduction*)).tw. or agreement.tw. or precision.tw. or imprecision.tw. or precise values.tw. or test-retest.tw. or (test adj3 retest).tw. or (reliab* adj3 (test or retest)).tw. or stability.tw. or interrater.tw. or inter-rater.tw. or intrarater.tw. or intra-rater.tw. or intertester.tw. or inter-tester.tw. or intratester.tw. or intra-tester.tw. or interobserver.tw. or inter-observer.tw. or intraobserver.tw. or intra-observer.tw. or intertechnician.tw. or inter-technician.tw. or intratechnician.tw. or intra-technician.tw. or interexaminer.tw. or inter-examiner.tw. or intraexaminer.tw. or intra-examiner.tw. or interassay.tw. or inter-assay.tw. or intraassay.tw. or intra-assay.tw. or interindividual.tw. or inter-individual.tw. or intraindividual.tw. or intra-individual.tw. or interparticipant.tw. or inter-participant.tw. or intraparticipant.tw. or intra-participant.tw. or kappa.tw. or kappa's.tw. or kappas.tw. or repeatab*.tw. or ((replicab* or repeated) adj3 (measure or measures or findings or result or results or test or tests)).tw. or generaliza*.tw. or generalisa*.tw. or concordance.tw. or (intraclass adj3 correlation*).tw. or discriminative.tw. or known group.tw. or factor analysis.tw. or factor analyses.tw. or factor structure.tw. or factor structures.tw. or dimension*.tw. or subscale*.tw. or (multitrait adj3 scaling adj3 (analysis or analyses)).tw. or item discriminant.tw. or interscale correlation*.tw. or error.tw. or errors.tw. or individual variability.tw. or interval variability.tw. or rate variability.tw. or (variability adj3 (analysis or values)).tw. or (uncertainty adj3 (measurement or measuring)).tw. or standard error of measurement.tw. or sensitiv*.tw. or responsive*.tw. or (limit adj3 detection).tw. or minimal detectable concentration.tw. or interpretab*.tw. or ((minimal or minimally or clinical or clinically) adj3 (important or significant or detectable) adj3 (change or difference)).tw. or (small* adj3 (real or detectable) adj3 (change or difference)).tw. or meaningful change.tw. or ceiling effect.tw. or floor effect.tw. or Item response model.tw. or IRT.tw. or Rasch.tw. or Differential item functioning.tw. or DIF.tw. or computer adaptive testing.tw. or item bank.tw. or cross-cultural equivalence.tw. 6779036

3 atrial fibrillation/ or atrial fibrillat*.tw. or atrium fibrillat*.tw. or auricular fibrillat*.tw. 89359

4 1 and 2 and 3 1224

5 (delphi-technique or cross-sectional).ti. or addresses.pt. or biography.pt. or case reports.pt. or comment.pt. or directory.pt. or editorial.pt. or festschrift.pt. or interview.pt. or lectures.pt. or legal cases.pt. or legislation.pt. or letter.pt. or news.pt. or newspaper article.pt. or patient education handout.pt. or popular works.pt. or congresses.pt. or consensus development conference.pt. or consensus development conference, nih.pt. or practice guideline.pt. 4475005

6 4 not 5 1183

7 exp animals/ not humans/ 4867563

8 6 not 7 1181

**APPENDIX C: Full results from literature mapping exercise**

| **QoL Instrument** | **#** |
| --- | --- |
| SF-36 | 41 |
| Atrial Fibrillation Effect on QualiTy-of-Life (AFEQT) | 38 |
| EQ-5D (3L or 5L) | 24 |
| European Heart Rhythm Association (EHRA) | 13 |
| Canadian Cardiovascular Society Severity of Atrial Fibrillation (CCS-SAF) | 9 |
| University of Toronto Atrial Afibrillation Severity Scale (AFSS) | 8 |
| SF-12 | 8 |
| Hospital Anxiety and Depression Scale (HADS) | 7 |
| Minnesota Living with Heart Failure Instrument (MLHFQ) | 7 |
| Atrial fibrillation Quality of Life Instrument (AFQLQ) | 4 |
| Arrhythmia-Specific instrument in Tachycardia and Arrhythmia (ASTA) | 4 |
| Visual Analogue Scale (VAS) stress | 4 |
| AF Symptom Checklist, Frequency and Severity | 3 |
| State-Trait Anxiety Inventory (STAI) | 3 |
| Type D Scale-14 (DS-14) | 2 |
| Duke Activity Status Index (DASI) | 2 |
| Karnofsky Performance Status Scale | 2 |
| Montreal Cognitive Assessment (MoCA) | 2 |
| AF6 | 1 |
| AF-QoL | 1 |
| Anxiety Sensitivity Index (AnxSI-R) | 1 |
| Cardiac Anxiety Instrument | 1 |
| Center for Epidemiologic Studies Depression Scale (CES-D) | 1 |
| Control Attitudes Scale (CAS) | 1 |
| Disease Related Symptom Instrument | 1 |
| Fatigue Severity Scale | 1 |
| HeartQoL | 1 |
| Illness Perception Instrument | 1 |
| Knowledge of Atrial Fibrillation (KAF) | 1 |
| Mayo AF Symptom Index (MAFSI) | 1 |
| Multidimensional Fatigue Inventory (MFI-20) | 1 |
| New York Heart Association function class | 1 |
| Patient Activation Measure (PAM)-Short Form | 1 |
| Patient Assessment of Chronic Illness Care (PACIC) | 1 |
| Patient Health Instrument-9 | 1 |
| Perception of Anti‐Coagulant Treatment Instrument (PACT‐Q) | 1 |
| Pittsburg Sleep Quality Index | 1 |
| QL-form | 1 |
| Severity of AF (SAF) | 1 |
| SF-6 | 1 |
| Specific Symptom Scale | 1 |
| Stanford Presenteeism Scale | 1 |
| Symptoms Severity Instrument | 1 |
| WHOQOL-BREF | 1 |
| Work Productivity and Activity Impairment Instrument | 1 |

**APPENDIX D: Risk of bias and measurement property appraisal results**

**APPENDIX E: Data extraction table of measurement properties**
